# The impact of antiretroviral therapy on adult mortality in rural Tanzania

**DOI:** 10.1111/j.1365-3156.2011.02924.x

**Published:** 2012-07-30

**Authors:** Milly Marston, Denna Michael, Alison Wringe, Raphael Isingo, Benjamin D Clark, Aswile Jonas, Julius Mngara, Samweli Kalongoji, Joyce Mbaga, John Changalucha, Jim Todd, Basia Zaba, Mark Urassa

**Affiliations:** 1 London School of Hygiene and Tropical MedicineUK; 2 The National Institute of Medical Research, MwanzaTanzania; 3 Magu District CouncilMagu, Mwanza, Tanzania

**Keywords:** antiretroviral therapy, HIV, mortality, cohort, Tanzania

## Abstract

**Objective:**

To describe the impact of antiretroviral therapy (ART) on mortality rates among adults participating in an HIV community cohort study in north-west Tanzania.

**Methods:**

Serological and demographic surveillance rounds have been undertaken in a population of approximately 30 000 people since 1994. Free HIV care including ART has been available since 2005. Event history analysis was used to compare mortality rates among HIV-negative and HIV-positive adults in the 5-year period before and after the introduction of ART. Crude and adjusted hazard ratios were calculated using exponential regression models. Interaction between time period and HIV status was assessed to investigate whether there was a non-linear relationship between these two variables.

**Results:**

Male and female mortality patterns varied over the pre- and post-ART period. In women, the crude death rate fell for both HIV negatives and HIV positives hazard rate ratio (HRR = 0.71; 95%CI 0.51–0.99 and HRR = 0.68; 95%CI: 0.46–0.99, respectively). For men, the mortality among the HIV negatives increased (HRR = 1.47; 95%CI: 1.06–2.03) while the decline in mortality among the HIV positives (HRR = 0.77; 95%CI 0.52–1.13) was not statistically significant. The largest decrease in HIV-positive mortality over the two periods was among the 30- to 44-year-old age group for women and among the 45- to 59-year-old age group for men.

**Conclusion:**

There has been a modest effect on mortality in the study population following the introduction of free ART 5 years ago. Improving access to treatment and placing greater focus on retaining individuals on treatment are essential if the full potential of treatment for reducing HIV-related mortality is to be realised.

## Background

In recent years, the availability of antiretroviral therapy (ART) has dramatically increased in sub-Saharan Africa, with 4 million people estimated to be receiving treatment by the end of 2008 (WHO 2009), representing an increase of 300% from December 2005 (WHO 2006). In Tanzania, national-level estimates of ART coverage, defined as the proportion of those in need of treatment who were receiving ART, increased from 18% to 31% between 2006 and 2007 (WHO 2008), although these figures mask substantial regional variations (Somi *et al.* 2011a).

Despite the apparent success in bringing HIV treatment to millions of Tanzanians, the national programme has faced numerous challenges, particularly in terms of achieving high rates of patient retention after the initiation of treatment. Data from 88 817 people attending 101 ART clinics in Tanzania suggest that the cumulative proportion of patients who are no longer in follow-up within 2 years of treatment initiation is 40% for men and 34% for women (Somi *et al.* 2011b). Among those with known outcomes, mortality is strongly associated with CD4 count at treatment initiation, with those starting ART with a CD4 count of <50/μl having 2.19 (95%CI: 2.08–2.67) times the hazard of death compared with those starting treatment with a CD4 count between 50 and 200 U/l (Somi *et al.* 2011b).

One of the most important measures of the success of an ART programmes is its impact on HIV-related mortality at the population level. Studies in Malawi and Ethiopia have documented declines in mortality after the introduction of ART, using death registers, coffin sales and burial surveillance (Reniers *et al.* 2009; Mwagomba *et al.* 2010).

HIV community-level cohort studies that include demographic surveillance systems (DSS) and verbal autopsy data are even better placed to directly measure the impact of ART on mortality in the population from the time that treatment first became available. In a DSS study in the north of Malawi, declines in the overall mortality rates among adults were observed within 8 months of free ART becoming available, with mortality reduced by 35% (adjusted rate ratio = 0.65; 0.46–0.92) in adults living near the main road, where mortality prior to ART availability had been highest (Jahn *et al.* 2008). A later study in the same setting detected a decline of 32% in the AIDS-specific mortality rate in the 2 years after the introduction of ART at a clinic in the study area, compared with the 3 years before, with estimated treatment coverage of approximately 70% (Floyd *et al.* 2010). In a South African cohort study that incorporated DSS data, HIV-related age-standardised mortality declined significantly, from 22.5 to 17.6 per 1000 person-years in women 25–49 years of age (*P* < 0.001) and from 26.5 to 18.7 per 1000 person-years in men 25–49 years of age (*P* < 0.001) between 2002 and 2003, before ART availability, and 2004–2006, after ART was introduced (Herbst *et al.* 2009).

In Kisesa in the north-west of Tanzania, an open HIV cohort study has been monitoring the dynamics of the HIV epidemic since 1994. In this setting, the proportion of the HIV-infected population in need of ART has been estimated as 26% in men and 23% in women in 2004, just before the start of the national HIV treatment programme (Zaba 2012), with coverage of treatment at 3% by the end of 2007, 2 years after the introduction of free ART in the regional referral hospital through the national HIV treatment programme (Wringe *et al.* 2012), since then coverage has increased with the introduction in 2008 of ART in the local health centre. The aim of this paper was to measure the impact of antiretroviral therapy provision on trends in adult mortality in this setting.

## Methods

### Study setting

The Kisesa cohort study has been monitoring the dynamics of the HIV epidemic since 1994 through regular serological, behavioural and demographic surveillance among a population of approximately 30 000 individuals living in six rural villages and a trading centre in a rural area of north-west Tanzania. The study area is located approximately 20 km from Mwanza, the country’s second city, bordering Lake Victoria. Most people are subsistence farmers and economic activities include petty trade of milk and vegetables in the trading centre (Mwaluko *et al.* 2003). There are six health facilities in the study area, including one health centre in the trading centre.

### Cohort study methods

Demographic rounds, collecting information on births, deaths and migration, take place at each household approximately twice per year as part of the DSS. Serological surveillance rounds take place within each village approximately every 3 years among consenting adults who were resident in the ward at the preceding demographic surveillance round. The serological surveillance rounds include HIV testing for research purposes without results disclosure and a detailed questionnaire on fertility, sexual behaviour and use of health services. Participants have access to a free health clinic for themselves and all family members, and since the 2000–2001 round, they can undergo voluntary counselling and testing (VCT) if they wish to know their HIV status.

A dried blood spot taken from a finger prick is tested for HIV at the National Institute of Medical Research in Mwanza, using Vironostika HIV-MIXT and Enzygnost HIV1/HIV2. Samples are considered to be HIV positive if both ELISA results are reactive and any discrepant results are re-tested by repeating the two ELISA tests. Any samples still returning a discrepant result are excluded from analysis.

Participation at the serological surveys has declined slightly over time from 74% to 61% between 1994/5 and 2006/7 (Urassa *et al.* 2010). HIV prevalence at the 2006–2007 serological surveillance round was 6% among men and women. HIV incidence was 1.1% in 2000–2003 (Wambura *et al.* 2007).

### Provision of HIV services

A permanent VCT service has been available to the study population since early 2005 at the health centre in the trading centre. In addition, temporary VCT services have been provided in each village during the serological surveys. VCT uptake during the serological surveys was <1% in 2000–2001, 10% in 2003–2004 and 17% in 2006–2007 (Isingo 2012).

Free HIV care and treatment, including ART, first became available to the study population at the beginning of 2005 through the national HIV programme. HIV care was initially available at the zonal and regional referral hospitals in Mwanza and was subsequently rolled out to district hospitals and health centres. Free ART has been available at the Kisesa health centre in the trading centre since September 2008. Individuals receiving a positive HIV diagnosis at the VCT centre in the health centre or the serological surveys are referred to the closest ART clinic for a clinical and immunological assessment to determine their eligibility to start HIV treatment. Money is provided to cover transportation costs from the Kisesa health centre to the ART clinic in Mwanza and diagnosed persons are linked to a local home-based care organisation for additional support if they wish (Nsigaye *et al.* 2009).

Data from referral forms, along with attendance data from the VCT services and the ART clinic, have been anonymously linked to the cohort data since 2005, enabling the number of HIV-infected individuals in the study population who initiated ART between January 2005 and December 2009 to be estimated.

### Statistical analysis

Subjects entered into follow-up at the date of their participation in the first serological surveillance round, or the date that they entered the cohort through the DSS, if they did not attend the first sero-survey. Exit was because of out-migration, censoring at the last DSS round they were seen or death, the last round used took place at the end of 2009. Subjects who entered the cohort after the 2006–2007 serological surveillance round were excluded from the analysis, as they would not yet have had an opportunity to undergo HIV testing.

Person-years of follow-up were divided into the following HIV status groups: (i) HIV infected, (ii) HIV negative, (iii) unknown (for those never tested, prior to first test, and more than 5 years after the last negative HIV test). The HIV-positive group contained the person-years of individuals after their first positive test. The HIV-negative group contained all the person-years of follow-up for individuals known to be HIV negative, as well as the person-years in a fixed time interval following a negative HIV test (5 years). It is essential to include a segment of time following on from the last negative test, since otherwise no deaths could be observed among the HIV negative, as their exposure time would end at their last HIV test when they are known to be still alive. The cut-off time selected for the post-negative group was taken as 5 years, after that their HIV status was classified as unknown. For sero-converters (613), a mid-point between the last negative and first positive test was taken and the person-years assigned accordingly.

Person-years were further split over two time periods: (i) the 5 years prior to the introduction of ART and (ii) the period after the introduction of ART. The introduction of ART in Kisesa was taken as 3 March 2005.

Crude and adjusted hazard ratios were calculated using exponential regression models. Interaction terms were used between time period and HIV status to investigate whether there was a non-linear relationship between the two variables. All analyses were performed using Stata 11.1.

### Ethical clearance

Ethical approval for the study was granted by the Tanzanian Medical Research Coordinating Committee and the London School of Hygiene and Tropical Medicine.

## Results

### Overall mortality

Overall, the crude adult mortality rate among 15–59-year olds declined by 17% (hazard rate ratio (HRR) = 0.83; 95%CI: 0.72–0.95) between the pre-ART and the post-ART periods. This change in mortality over the two time periods was predominantly because of a fall in female mortality from 8.8 deaths per 1000 person-years to 6.5 deaths per 1000 person-years (HRR = 0.73; 95%CI: 0.60–0.89). The crude male mortality rate remained similar over the two time periods at 9.1 in the pre-ART period and 8.5 in the post-ART period (Table 1). Over the whole time period, the crude mortality rate in those who are HIV positive is very high compared with those who are HIV negative, with a hazard rate ratio of 11.4 (95% CI: 8.9–14.7) for men and 9.4 (95% CI: 7.4–12.12) for women.

**Table 1:** Distribution of person-years and deaths with crude hazard rates

	Men						Women					
		
				Crude hazard ratios						Crude hazard ratios		
								
	Deaths	Person-years (per 1000)	Death rate (per 1000)	HRR	(95% CI)	*P*-values	Deaths	Person-years (per 1000)	Death rate (per 1000)	HRR	(95% CI)	*P*-values
Period												
Pre-ART	260	28.56	9.1	1			263	29.75	8.84	1		
Post-ART	182	21.5	8.47	0.93	(0.77–1.12)	0.452	151	23.36	6.47	0.73	(0.60–0.89)	0.002
HIV status												
Negative	149	26.82	5.56	1			150	31.03	4.83	1		
Positive	106	1.68	63.11	11.36	(8.86–14.57)	<0.001	111	2.43	45.64	9.44	(7.39–12.07)	<0.001
Unknown	187	21.56	8.67	1.56	(1.26–1.94)	<0.001	153	19.64	7.79	1.61	(1.29- 2.02)	<0.001
Age group												
15–29	112	27.23	4.11	1			117	26.49	4.42	1		
30–44	194	15.16	12.8	3.11	(2.47–3.93)	<0.001	187	17.74	10.54	2.39	(1.89–3.01)	<0.001
45–59	136	7.67	17.73	4.31	(3.36–5.54)	<0.001	110	8.87	12.4	2.81	(2.16–3.64)	<0.001
Residence												
Rural	234	26.99	8.67	1			205	27.82	7.37	1		
Trading centre	97	10.13	9.58	1.1	(0.87–1.40)	0.409	97	11.31	8.57	1.16	(0.91–1.48)	0.219
Roadside	111	12.93	8.58	0.99	(0.79–1.24)	0.933	112	13.97	8.02	1.09	(0.86–1.37)	0.472

ART, antiretroviral therapy; HRR, hazard rate ratio.

Pre-ART = before 1st Jan 2005.

Post-ART = after 1st Jan 2005.

### Mortality by HIV status

Male and female trends in mortality vary over the pre- and post-ART period (Tables 2 and 3). In women, the crude death rate fell for both HIV negatives and HIV positives (HRR = 0.71; 95%CI 0.51–0.99 and HRR = 0.68; 95%CI: 0.46–0.99, respectively). For men, the mortality among the HIV negatives increased (HRR = 1.47; 95%CI: 1.06–2.03) while the statistical evidence for a change in mortality among the HIV positives was inconclusive (HRR = 0.77; 95%CI 0.52–1.13). The largest decrease in HIV-positive mortality over the two periods was among the 30- to 44-year-old age group for women (HRR = 0.51, 95% CI 0.31–0.86) and among the 45- to 59-year-old age group for men (HRR = 0.46, 95% CI 0.23–0.93).

**Table 2:** Crude hazard rate ratios in adults aged 15–59 pre- and post-ART roll-out and HIV status for men

	Men – HIV negative						Men – HIV positive					
		
	Death	Person-years per 1000	Death rate	Crude RR	95% CI	*P*-value	Death	Person-years per 1000	Death rate	Crude RR	95% CI	*P*-value
ART period												
Pre-0–5 years	62	13.71	4.52	1			63	0.89	70.77	1		
Post-1st period	87	13.11	6.64	1.47	(1.06–2.03)	0.021	43	0.79	54.48	0.77	(0.52–1.13)	0.186
Age grouped												
15–29												
Pre-0–5 years	10	7.38	1.35	1			10	0.31	31.96	1		
Post-1st period	26	7.26	3.58	2.64	(1.27–5.48)	0.009	7	0.22	31.92	1.00	(0.38–2.62)	0.998
30–44												
Pre-0–5 years	23	4.26	5.4	1			30	0.42	71.49	1		
Post-1st Period	30	3.6	8.33	1.54	(0.90–2.65)	0.118	24	0.39	61.32	0.86	(0.50–1.47)	0.575
45–59												
Pre-0–5 years	29	2.07	13.99	1			23	0.16	145.9	1		
Post-1st period	31	2.24	13.83	0.99	(0.60–1.64)	0.964	12	0.18	67.19	0.46	(0.23–0.93)	0.029
Area of residence												
Rural												
Pre-0–5 years	35	8.93	3.92	1			30	0.47	64.01	1		
Post-1st period	58	7.95	7.3	1.86	(1.22–2.83)	0.004	23	0.43	53.54	0.84	(0.49–1.44)	0.519
Urban												
Pre-0–5 years	19	2.57	7.4	1			14	0.2	68.48	1		
Post-1st period	14	2.62	5.35	0.72	(0.36–1.44)	0.356	10	0.18	54.94	0.80	(0.36–1.81)	0.595
Roadside												
Pre-0–5 years	8	2.22	3.61	1			19	0.22	87.53	1		
Post-1st period	15	2.54	5.91	1.64	(0.69–3.86)	0.260	10	0.18	56.28	0.64	(0.30–1.38)	0.258

ART, antiretroviral therapy.

**Table 3:** Crude hazard rate ratios in adults aged 15–59 years in the pre- and post-ART periods and HIV status, for women

	Women – HIV negative						Women – HIV positive					
		
	Death	Person-years per 1000	Death rate	Crude RR	95% CI	*P*-value	Death	Person-years per 1000	Death rate	Crude RR	95% CI	*P*-value
ART period												
Pre-0–5 years	89	15.81	5.63	1			65	1.19	54.69	1		
Post-1st period	61	15.22	4.01	0.71	(0.51–0.99)	0.041	46	1.24	36.99	0.68	(0.46–0.99)	0.042
Age grouped												
15–29												
Pre-0–5 years	17	6.67	2.55	1			18	0.51	35.3	1		
Post-1st period	14	6.31	2.22	0.87	(0.43–1.77)	0.701	10	0.37	27.28	0.77	(0.36–1.67)	0.513
30–44												
Pre-0–5 years	36	6.08	5.92	1			38	0.55	69.44	1		
Post-1st period	24	5.66	4.24	0.72	(0.43–1.20)	0.206	24	0.67	35.66	0.51	(0.31–0.86)	0.011
45–59												
Pre-0–5 years	36	3.06	11.77	1			9	0.13	68.49	1		
Post-1st period	23	3.25	7.07	0.60	(0.36–1.01)	0.056	12	0.2	58.89	0.86	(0.36–2.04)	0.732
Area of residence												
Rural												
Pre-0–5 years	54	9.52	5.67	1			27	0.52	52.06	1		
Post-1st period	32	8.51	3.76	0.66	(0.43–1.03)	0.065	16	0.52	30.55	0.59	(0.32–1.09)	0.091
Urban												
Pre-0–5 years	14	3.23	4.34	1			15	0.25	59.59	1		
Post-1st period	14	3.35	4.19	0.96	(0.46–2.02)	0.924	18	0.35	51.71	0.87	(0.44–1.72)	0.685
Roadside												
Pre 0–5 years	21	3.07	6.85	1			23	0.42	55	1		
Post-1st period	15	3.37	4.45	0.65	(0.34–1.26)	0.203	12	0.37	32.3	0.59	(0.29–1.18)	0.135

ART, antiretroviral therapy.

The exponential regression models (Table 4) show that for men, the change in mortality over time is different for those who were HIV positive compared with those who were HIV negative with an interaction term between the time period and HIV status of 0.48 (95% CI 0.29–0.80). This means that while mortality among HIV-negative men rose slightly (HRR = 1.48; 95%CI: 1.07–2.05), among HIV-positive men, there was evidence of borderline significance to suggest that mortality declined (HRR = 0.72; 95%CI: 0.48–1.06). Among women, there was no evidence of interaction between the time period and HIV status. The adjusted mortality rate was 13.6 (95% CI 9.5–19.3) times higher for men who were HIV positive compared with those who were HIV negative and 10.4 times higher for women (95%CI 7.5–14.3) over the entire period 2000–2009 (Table 4).

**Table 4:** Adjusted mortality hazard rate ratios using exponential regression by sex

	Males			Females		
		
	Hazard ratio	95% CI	*P*-values	Hazard ratio	95% CI	*P*-values
Period						
Pre-ART	1			1		
Post-ART	1.48	(1.07–2.05)	0.019	0.70	(0.51–0.97)	0.034
HIV status						
Negative	1			1		
Positive	13.57	(9.54–19.32)	<0.001	10.35	(7.49–14.30)	<0.001
Unknown	2.33	(1.71–3.16)	<0.001	2.00	(1.50–2.66)	<0.001
Interaction between period and HIV status						
Negative post-ART to pre-ART	1			1		
Positive post-ART to pre-ART	0.48	(0.29–0.80)	0.005	0.85	(0.52–1.40)	0.523
Unknown post-ART to pre-ART	0.45	(0.28–0.71)	0.001	0.94	(0.58–1.51)	0.793
Area of residence						
Rural	1			1		
Roadside	0.97	(0.76–1.23)	0.784	1.01	(0.80–1.29)	0.908
Trading centre	0.86	(0.68–1.08)	0.189	0.90	(0.71–1.13)	0.352
Age group						
15–29	1			1		
30–44	2.66	(2.10–3.36)	<0.001	2.34	(1.85–2.97)	<0.001
45–59	3.92	(3.04–5.04)	<0.001	3.29	(2.52–4.29)	<0.001

ART, antiretroviral therapy.

### ART uptake

Antiretroviral therapy uptake among the study population steadily increased between 2005 and 2009: the number of men on treatment doubled and the number of women quadrupled (Table 5). ART uptake in 2009 is highest for men and women aged between 30 and 44 years old, although the distribution is skewed towards the younger ages for women and towards the older ages for men. There is no evidence of a difference between men and women in the CD4 count or WHO stage at the time of treatment initiation.

**Table 5:** Characteristics of adults in the study population on ART, at treatment initiation, by sex

	Men		Women	
		
	*N*	%	*N*	%
Age group				
15–29	29	13.18	150	32.05
30–44	109	49.55	233	49.79
45–59	48	21.82	51	10.90
WHO stage				
1	34	15.45	55	11.75
2	38	17.27	93	19.87
3	46	20.91	75	16.03
4	9	4.09	27	5.77
CD4 group				
<50	26	11.82	47	10.04
50–199	71	32.27	137	29.27
200+	26	11.82	62	13.25
Year				
2005	29	13.18	41	8.76
2006	30	13.64	69	14.74
2007	42	19.09	88	18.80
2008	55	25.00	116	24.79
2009	64	29.09	154	32.91
Total	220	100.00	468	100.00

ART, antiretroviral therapy.

## Discussion

Adult mortality in Kisesa generally fell between 2000 and 2010, which is largely attributable to the decline in deaths among women. There was some evidence that ART uptake among the study population has had an impact on adult mortality among men, since despite a slight increase in the mortality rates among the HIV negatives, there was no equivalent rise in the mortality among the HIV positives, with some suggestion that it had decreased. These differences were not observed among the women, where mortality dropped among both the HIV positives and the HIV negatives by a similar degree. The mortality of men with unknown HIV status appeared to fall dramatically, which is most likely due to the changing composition of the group. It is plausible that with the availability of ART in the area, the incentive to participate in the serological survey has become greater, particularly for those who suspect that they may be infected with HIV, as VCT services can be accessed within the village. In this case, over time, a growing proportion of individuals with an unknown HIV status will actually be HIV-negative individuals, and these persons will have lower mortality over the pre- and post-ART periods.

Other HIV cohort studies in sub-Saharan Africa that began ART roll-out at around the same time have found stronger evidence of an impact on mortality (Herbst *et al.* 2009; Floyd *et al.* 2010; Kasamba *et al.* 2012). Nevertheless, there are important differences between each setting, particularly in terms of access to treatment and the speed at which decentralisation of services occurred. For residents in Kisesa, ART was initially only available via a referral from the local VCT clinics to the zonal referral hospital in Mwanza City, approximately 20 km away. Several qualitative studies identified the long journey time, waiting times at the clinic and a lack of familiarity with the city and intimidation at the very large health facility as barriers to attending the ART clinic at the hospital (Nsigaye *et al.* 2009; Roura *et al.* 2009; Wringe *et al.* 2009), explaining the low ART coverage observed by the end of 2007 (Wringe *et al.* 2012). Although ART treatment became available in the Kisesa health centre in September 2008 as part of the government’s plans to decentralise ART, the mortality impact from the increasing number of persons initiating treatment is unlikely to be seen immediately. In the other studies, ART treatment was available to participants locally in the study area, either from the beginning or shortly after the introduction of HIV treatment by the national programme, which is likely to have resulted in a much higher coverage of ART within a shorter time-frame, enabling the effects of treatment on mortality to be observed more quickly (Wringe *et al.* 2012).

This study showed that in those where the HIV status was known, 42% of the deaths (111/261 – 43% in women, and 106/255 – 42% in men) occurred in those who were HIV positive, despite the fact they represented <10% of the population, and only 6.6% of the person-years. In the period before ART, 46% (128/279) of all deaths occurred in HIV positive, which reduced to 38% (89/337) following the introduction of ART. Overall, this corresponded to 20 deaths averted in the period following ART introduction.

Bigger changes in mortality in Kisesa may have occurred through changes in the environment in the ward. In the last 7 years, the road from Mwanza to Kenya has been tarmaced, and Kisesa has changed from a quiet village producing cotton to a centre for trading rice, and a dormitory suburb of Mwanza city. This new traffic may account for some of the increased number of deaths in young men.

For this analysis, we assumed that a person remains HIV negative for 5 years after the last negative HIV test. Changing this assumption to 2 years after the last negative HIV test lowered the mortality level slightly giving 4.1 and 3.0 deaths per 1000 person-years pre- and post-ART for women compared with 5.6 and 4.0 when using 5 years. Similar results were found for men with the changed assumption. Similarly, inclusion of a period of 1 year prior to the first positive HIV test in the calculation of the HIV positive mortality rates lowered the estimates slightly, giving mortality rates of 65.7 and 50.5 per 1000 person-years, pre- and post-ART, for positive men compared to 70.8 and 54.5 when excluding the period prior to the first positive HIV test. This accords with the previously reported low mortality in the first 2 years following sero-conversion (Todd *et al.* 2007).

In conclusion, there has been a modest effect on mortality in the study population following the introduction of free ART 5 years ago. Improving access to treatment and placing greater focus on retaining individuals on treatment are essential if the full potential of treatment for reducing HIV-related mortality is to be realised.
